# Single‐cell marker gene clustering: A unified deep learning framework for marker gene‐based clustering of single‐cell RNA‐sequencing data

**DOI:** 10.1002/qub2.70047

**Published:** 2026-07-24

**Authors:** Shahriar Rahman Niloy, Toushif Muktashid Hasan, Md. Saiduzzaman Apu, Fahim Hafiz, Salekul Islam, Riasat Azim

**Affiliations:** ^1^ Department of Computer Science and Engineering United International University Dhaka Bangladesh; ^2^ Department of Electrical and Computer Engineering North South University Dhaka Bangladesh

**Keywords:** clustering, deep learning, marker genes, optimization, scRNA‐seq

## Abstract

Single‐cell RNA sequencing (scRNA‐seq) has transformed the study of cellular heterogeneity by making it possible to classify individual cells and their functional states. However, the analysis remains difficult because high dropout rates lead to sparse and noisy expression data. Existing marker gene selection methods are often fragmented, typically relying on a single strategy that overlooks complementary biological signals. Without a unifying framework for denoising and marker identification, clustering can suffer in accuracy, stability, and interpretability. This makes it more difficult to define cell subpopulations and extract meaningful biological insights clearly. We introduce single cell marker gene clustering (scMGC), a unified framework that integrates a denoising autoencoder with multi‐method marker selection for scRNA‐seq clustering. The autoencoder reduces dropout noise, whereas a unified marker gene scoring system balances the contributions of multiple methods to select reliable markers. These markers are then applied in graph‐based clustering to uncover accurate and interpretable cell subpopulations. scMGC was benchmarked against seven state‐of‐the‐art methods across seven scRNA‐seq datasets. On average, it outperformed competing approaches by 31.3% in adjusted rand index and 28.2% in normalized mutual information, showing consistent improvements in clustering accuracy. Enrichment and disease association analyses further validated that the discovered clusters are both biologically meaningful and clinically relevant. The codes and datasets used are available on the GitHub website (srniloy/scMGC).

## 1 INTRODUCTION

In recent years, single‐cell analysis techniques, such as single‐cell RNA sequencing (scRNA‐seq), allow for the characterization of cell states and special functions that may profoundly impact subsequent research. This advancement has catalyzed the development of sophisticated cell clustering techniques. Meanwhile, traditional approaches are effective in classifying scRNA‐seq in varying degrees. However, although sequencing data obtained from such techniques provides stable estimates of cell‐type heterogeneity over a widening array of biological settings, thus maximizing the potential of single‐cell sequencing. Another big challenge remains when scRNA‐seq datasets are marred by a very high dropout rate. In these cases, a majority of expressed transcripts cannot be detected. Hence, the matrix that is produced becomes sparse and noisy. This obscures actual biological signals, as shown in Figure 1. Consequently, reducing the problem to a small but functionally meaningful set of genomic determinants driving this heterogeneity is an inherently difficult problem, often making computational approaches infeasible.

**FIGURE 1:**
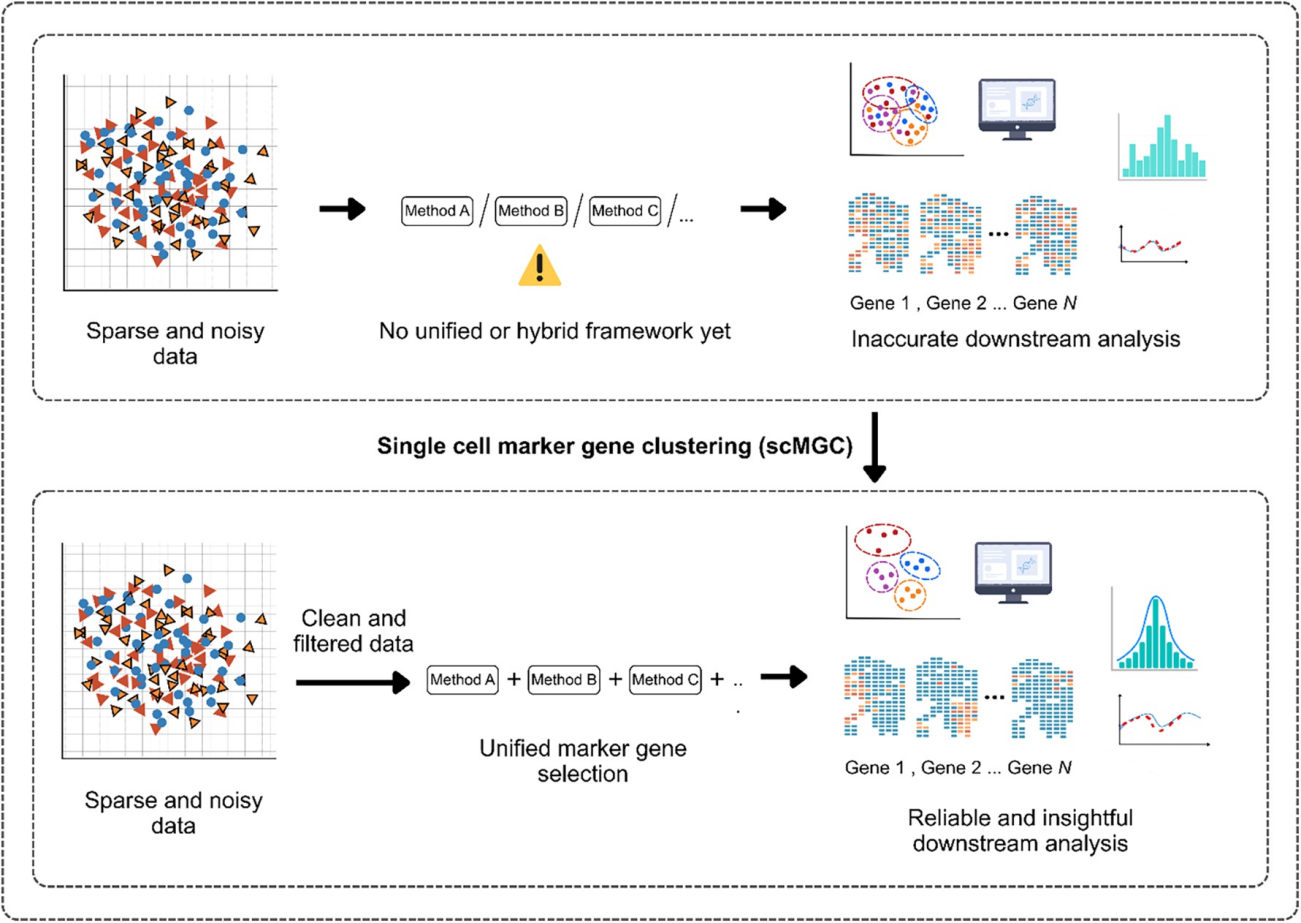
Illustration of the need for a unified marker gene selection strategy to improve downstream analyses in scRNA‐seq. Existing methods select marker genes from high‐dimensional feature spaces using diverse computational approaches. Yet often fail to capture the full distributional characteristics of marker genes. Our proposed method, single‐cell marker gene clustering, integrates multiple state‐of‐the‐art selection techniques into a unified pipeline. Enabling the discovery of an optimal marker gene set that enhances downstream reliability and clustering performance.

Recognizing distinct cell types is essential for interpreting the functional units of an organism within complex biological systems [1]. The advancement of scRNA‐seq analysis has transformed biological studies, enabling tasks such as building cellular trajectories, detecting differentially expressed genes (DEG), and characterizing the boundaries of individual cell subgroups [2]. However, a significant gap remains in the availability of a scalable and generalizable method for selecting marker genes. This deficiency hinders our ability to accurately delineate distinct subpopulations of cells, which is essential for the design of gene expression for transcriptomics. Consequently, there is a pressing need for a method that can effectively denoise the expression data to recover reliable signals before attempting to identify defining features. Given that a multitude of scRNA‐seq methodologies continue to hinge predominantly upon the stratification of cellular populations into discrete classifications as their foundational analytical paradigm [3]. Additionally, the methodology segments into two distinct methods of marker gene‐specific identification from scRNA‐seq data: the careful curation of gene lists based on expert knowledge and the use of differential expression analysis (DEA). Yet, when implemented in large and complex networks, such algorithms tend to encounter local optimum‐related issues that may adversely impact their capacity to yield optimal clustering results.

There have been many computational methods aimed at improving cell type identification and classification from scRNA‐seq data. One of them is Wang et al., who introduced CHLNMF [4]. This method uses a Cauchy loss function instead of the traditional Euclidean distance within the non‐negative matrix factorization (NMF) framework. This replacement helps eradicate the effects of noise. CHLNMF also employs hypergraph regularization to maintain high‐order structural relationships among cells. This provides better clustering accuracy and biologically more relevant cell‐type groupings. It is one of the strengths of this algorithm that it can quickly determine the optimal number of clusters and thus enable precise cell‐type identification. However, it may not always achieve a global optimum due to the employment of half‐quadratic optimization. This is true when applied to complex or noisy datasets.

Huang et al. proposed a high‐order element‐wise weighting strategy within a self‐representative ensemble learning framework, termed scEWE [5]. This approach assigns distinct base clustering weights to individual cells. These weights facilitate the careful construction and optimization of the consensus matrix. Furthermore, high‐order information between cells is extracted, enhancing the representation of similar relationships among them.

Wang et al. presented scHFC [6], a hybrid fuzzy clustering technique that combines fuzzy C‐means (FCM) with the Gath–Geva (GG) algorithm, further refined via natural computation. The method uses principal component analysis (PCA) to make scRNA‐seq data less complex. Then, the enhanced FCM makes a membership matrix that helps the GG algorithm make the final clustering results. They also suggested a multi‐index comprehensive estimation strategy that uses four clustering effectiveness indices to find the best number of clusters. Simultaneously, Zhu et al. proposed scMEB [7], a method for finding DEG across different cell types and unlabeled samples that does not depend on clustering. scMEB cuts down on false negatives in DEG detection and ranks genes by minimizing the enclosing ball of stably expressed genes. It also ranks genes based on their distance from the center of the sphere. The use of PCA makes the method even more accurate and efficient. In addition to these methods, Missarova et al. proposed geneBasis [8], a computational framework that finds useful gene subsets from scRNA‐seq data. GeneBasis allows clustering and visualizing data with small but biologically important gene sets while keeping the diversity and structure of cells.

Wang et al. [9], on the other hand, differ from traditional low‐rank representation (LRR) techniques by dynamically adjusting their dictionary matrix during optimization. This adaptability makes it well‐suited for scRNA‐seq data, allowing direct subspace clustering without relying on spectral clustering. Consequently, it reduces noise interference and enhances cell‐type annotation accuracy. Nonetheless, the high noise levels and significant missing values in scRNA‐seq data pose challenges. Using the raw data as the basis for constructing the low‐dimensional subspace representation may hinder the accuracy of the LRR matrix. Furthermore, the clustering method used may not align with the learned affinity matrix, limiting the ability to achieve optimal clustering results.

Shu et al. introduced scMMN [10], a deep learning framework that amalgamates contrastive learning and graph‐based methodologies to generate multi‐view representations of scRNA‐seq data. By using k‐nearest neighbors (kNN), diffusion mapping, and graph Laplacian filters together, scMMN improves clustering performance, especially in cross‐species datasets and complex tissue structures. It also shows big gains in scalability and flexibility compared to traditional graph‐based frameworks. Yin et al. also created CellTICS [11], an interpretable neural network for identifying cell types and sub‐cell types. It uses Reactome pathway hierarchies to make biological data easier to understand. CellTICS uses a multi‐predictive‐layer strategy to get better results and show important pathways that explain expression stochasticity. This is better than other methods and gives new information about cellular heterogeneity in situations such as aging and disease.

Zhang et al. introduced a feature selection method based on gene expression decomposition (FEED) [12], which utilizes a gene correlation calculation technique to assess the relationships between genes from a distributional perspective. Following this, a permutation‐based approach is employed to establish the threshold for gene importance, allowing for the identification of marker gene subsets. Consequently, genes with high scores are retained as marker genes.

Vlot et al. introduced SEMITONES [13], a method for identifying markers in single‐cell omics without clustering. It recognizes features that are enriched in neighborhoods of cells based on continuous developmental trajectories and distance‐based sampling, such as radial basis function kernel on uniform manifold approximation and projection (UMAP). The software circumvents predefined clusters by using linear regression to test feature enrichment significance on shuffled data.

Nelson et al. developed SMaSH [14], a scalable framework for the selection of marker genes in scRNA‐seq data utilizing supervised learning techniques. SMaSH uses ensemble models such as XGBoost or deep neural networks with Shapley value attribution to rank genes by their classification value. This makes it possible to find cell‐type and tissue‐specific markers accurately. It has been demonstrated to surpass conventional linear approaches in terms of both accuracy and specificity, even with highly granular datasets. Its compatibility with the ScanPy framework renders it widely applicable to subsequent tasks, including spatial transcriptomics. Dai et al. also came up with COSG [15], a method based on cosine similarity that is fast and accurate for finding marker genes in many single‐cell modalities. COSG quantifies gene expression specificity in relation to an idealized cell‐type‐specific marker, demonstrating resilience to variations in scale and noise. It is much more accurate and faster than traditional statistical methods, and it works well even with datasets with millions of cells. It can also be used for scATAC‐seq and spatial transcriptomics, which makes it useful for a wide range of applications in cell‐type annotation and biological discovery.

Our approach attempts to improve the existing clustering methods by integrating autoencoders and combining clustering techniques that can sufficiently optimize feature learning operations, as deep learning has systematically catalyzed finer accuracy over single‐cell studies. However, these technologies require the purification of cell populations before sorting. Moreover, large‐scale datasets require expensive computational resources and pose challenges in segregating adjacent cell cultures within tissues. Compared to the traditional methods, we considered three different frameworks for our benchmark analysis to evolve our pipeline: CELLTICS [11], COSG [15], and SMaSH [16]. The wider array of performance from other independent models that enhance learning capabilities yields a greater variation of performance across different data sets. Furthermore, the model leverages the knowledge acquired from each method to identify and list the specific marker genes that characterize cell type features. Every technique possesses unique strengths and weaknesses, demanding that the choice of a particular technique be tailored to the specific traits of the dataset and objectives of the analysis. By employing a graph‐based clustering strategy, our framework reduces the likelihood of becoming confined to local optima, thereby yielding more stable and consistent results compared to traditional clustering approaches. This substantially improves the ability of our method to cope with issues of scalability and convergence to local optima. Finally, it reduces the high computational demands often observed in widely adopted clustering techniques.

Despite the widespread use of full‐expression or highly variable gene representations, clustering performance in scRNA‐seq analysis is often hindered by high dimensionality, dropout noise, and the inclusion of genes that are weakly related to cell identity. Marker genes provide a biologically grounded alternative by capturing cell‐type‐specific transcriptional programs that directly define cellular phenotypes. By focusing on genes that are consistently expressed within cell types while remaining distinct across populations, marker gene‐based representations enhance the signal‐to‐noise ratio and reduce the influence of irrelevant or noisy features. This property is particularly beneficial for graph‐based clustering methods, which rely on local similarity structures and are sensitive to noise and sparsity. Consequently, clustering driven by marker gene expression is expected to yield more coherent cell neighborhoods, improved cluster separability, and greater biological interpretability, motivating the design of unified frameworks, as shown in Figure 1 that explicitly integrate marker gene identification with downstream clustering.

To directly leverage the advantages of marker gene‐based representations while addressing sparsity, noise, and inconsistent feature selection, we introduce single‐cell marker gene clustering (scMGC), a new framework that greatly improves scRNA‐seq analysis through the combination of denoising autoencoders and selection of multi‐method markers used in the gene detection pipeline. A specialized denoising autoencoder first addresses high dropout rates by learning to impute missing values and suppress noise and yields a clean expression matrix. From these denoised data, scMGC then unifies three different marker gene selection methods to take advantage of each of their complementary strengths and produce a complete candidate gene set. An important component of our framework is the unified marker gene (UMG) scoring system. This system conducts feature selection by giving each method a dynamic weighting. The weight is a function of the method’s specific clustering performance. This allows the framework to intelligently combine each method into a single set of high‐confidence marker genes. This selected set of genes is then applied to graph‐based clustering to obtain precise and biologically relevant cell subpopulation identification. Through the unification of each of the pieces of our framework, scMGC successfully addresses the shortcomings of prior methods and represents a scalable, accurate, and interpretable solution to distinguishing cellular heterogeneity.

Moreover, our model was able to outperform similar applications when dealing with a wide variety of scRNA datasets. Through appropriate treatment of the diverse forms of noise while training, the model performs better on various datasets, thereby rendering it well‐suited for analyzing large‐scale scRNA‐seq data. We compare benchmarking with seven other models, which illustrates the overall superiority of our model across a diverse set of evaluation metrics. The experimental outcomes provide additional evidence that our framework effectively tackles major challenges in single‐cell data annotation, performing strongly in areas such as precise cell type identification and clustering. These results establish our approach as both dependable and efficient for supporting progress in single‐cell biology. The main contributions of this study are summarized as follows:

- Efficient integration of multiple advanced marker gene identification approaches.
- Marker gene‐based clustering that improves accuracy, stability, and biological interpretability in identifying cell subpopulations.
- Unified marker gene approach to further enhance the capability of standalone methods.
- Interpretability of the method demonstrated through downstream analysis, revealing strong correlations between marker genes and disease associations.

The rest of this paper is structured as follows. Section 2 discusses the experimental results and biological validations, Section 3 concludes with key findings and future research directions, and Section 4 presents the materials and methods of the proposed framework.

## 2 RESULTS

### 2.1 Clustering performance

We compared scMGC to eight marker–gene‐based methods across seven scRNA‐seq data sets (Biase, Goolam, Kolod, Kumar, Pollen, Robert, and Chu2). DUBStepR identifies a stable marker gene set by leveraging gene–gene correlations together with a Density Index that quantifies inhomogeneity in feature space [17]. Expr selects genes based on their overall expression level, following the baseline approach used in systematic clustering benchmarks [18]. FEAST ranks genes by iteratively performing initial clustering and estimating feature significance via an F‐statistic, refining the feature set through a validation step [19]. GiniClust detects both common and rare cell populations by selecting genes with a high Gini index, capturing skewed, sparsely expressed markers [20]. HVG selects highly variable genes relative to their mean expression, as implemented in the Seurat pipeline [21]. SCMarker performs ab initio marker gene selection using discriminative expression and mutual information criteria, without requiring predefined cell clusters [22]. Clustering performance was assessed using the adjusted rand index (ARI) [23] and the normalized mutual information (NMI) [24], which are widely accepted measures for evaluating predicted cluster assignments relative to reference labels. Figure 2 presents a side‐by‐side comparison of initial and scMGC clustering across seven benchmark datasets, showing that scMGC produces clearer boundaries and more biologically coherent groupings than the baseline. Tables 1 and 2 contain a numerical account of comparisons, whereas Figure 3 (ARI) and Figure 4 (NMI) include a visualization of findings.

**FIGURE 2:**
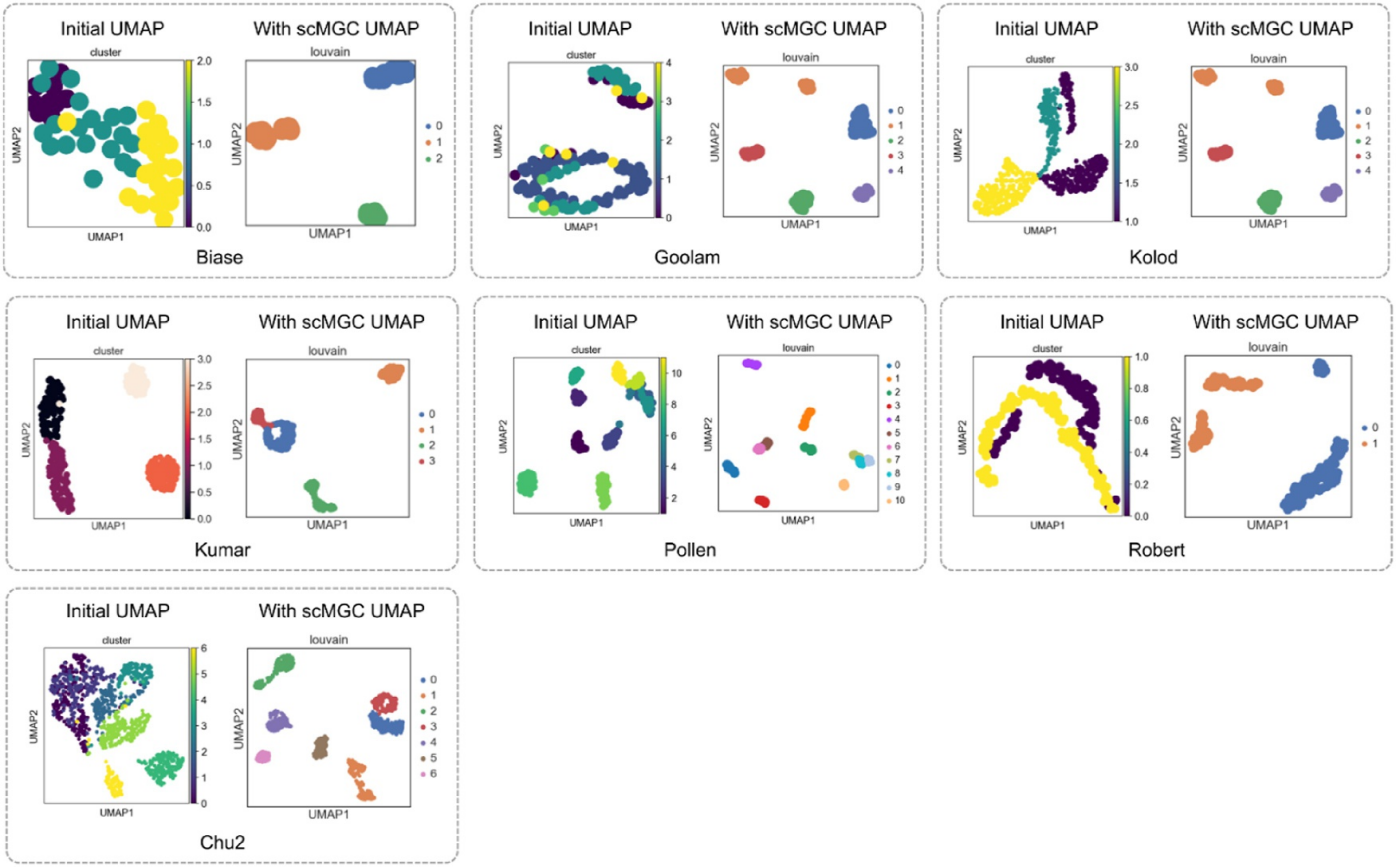
Comparison of initial clustering and single‐cell marker gene clustering (scMGC) with uniform manifold approximation and projection (UMAP) on seven gold standard datasets.

**TABLE 1:** Adjusted rand index (ARI) comparison of scMGC with eight competing clustering methods on seven datasets.

Dataset	DUBStepR	Expr	FEAST	GiniClust	HVG	SCMarker	geneBasis	FEED	scMGC	Improvement (%)^a^
Biase	NA	**0.48**	0.84	0.60	0.544	NA	0.006	**0.713**	1.00	19.0%
Goolam	0.273	0.265	0.159	0.279	0.214	0.34	**0.221**	**0.381**	0.54	41.7%
Kolod	0.051	0.023	0.024	0.022	0.023	0.021	**0.009**	**0.442**	0.58	31.2%
Kumar	0.172	0.651	0.674	0.633	0.70	**0.651**	0.704	**0.661**	0.744	5.7%
Pollen	0.787	0.698	0.797	**0.763**	0.814	0.785	0.804	**0.799**	0.956	17.4%
Robert	−0.002	0.001	0	0.001	**0.001**	0.449	−0.002	**0.403**	0.621	38.3%
Chu2	0.255	0.221	0.22	0.298	**0.476**	0.496	0.407	**0.38**	0.976	96.8%
**Overall improvement (%)** ^b^										**31.3%**

*Note*: NA, result not available for the corresponding method and dataset. Bold values indicate the best and second‐best results for each dataset.

^a^ Percentage improvement is computed as (s_ᵢ_ − b_ᵢ_)/b_ᵢ_ × 100, where sᵢ and bᵢ denote the ARI of scMGC and the second‐best method, respectively.

^b^ Overall percentage improvement is computed as Σ(s_ᵢ_ − b_ᵢ_)/Σb_ᵢ_ × 100.

**TABLE 2:** Normalized mutual information (NMI) comparison of scMGC with eight competing clustering methods on seven datasets.

Dataset	DUBStepR	Expr	FEAST	GiniClust	HVG	SCMarker	geneBasis	FEED	scMGC	Improvement (%)^a^
Biase	NA	**0.519**	0.828	0.607	0.595	NA	0.170	**0.736**	1.000	20.8%
Goolam	0.374	0.284	0.314	0.296	0.317	0.422	**0.360**	**0.447**	0.721	61.3%
Kolod	0.052	0.021	0.021	0.018	0.021	0.021	**0.019**	**0.431**	0.711	65.0%
Kumar	0.250	0.739	**0.769**	0.796	0.791	0.755	0.791	**0.772**	0.842	5.8%
Pollen	0.877	0.831	0.885	**0.853**	0.892	0.884	0.879	**0.879**	0.966	8.4%
Robert	0.009	0.011	0.009	0.014	**0.011**	0.450	0.009	**0.381**	0.609	35.3%
Chu2	0.471	0.386	0.384	**0.599**	0.699	0.692	0.577	**0.582**	0.978	40.3%
**Overall improvement (%)** ^b^										**28.2%**

*Note*: NA, result not available for the corresponding method and dataset. Bold values indicate the best and second‐best results for each dataset.

^a^ Percentage improvement is computed as (s_ᵢ_ − b_ᵢ_)/b_ᵢ_ × 100, where sᵢ and bᵢ denote the NMI of scMGC and the second‐best method, respectively.

^b^ Overall percentage improvement is computed as Σ(s_ᵢ_ − b_ᵢ_)/Σb_ᵢ_ × 100.

**FIGURE 3:**
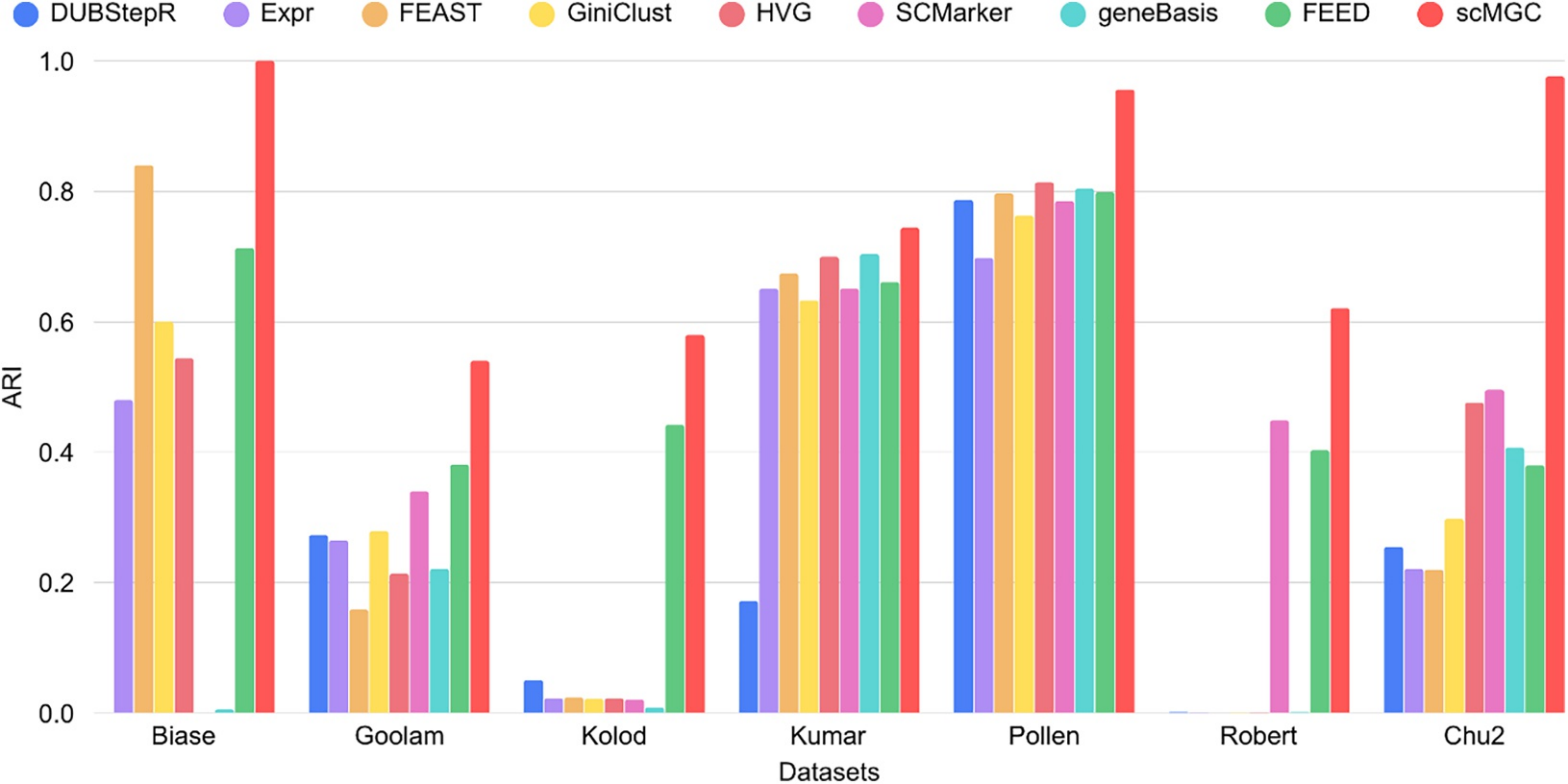
Adjusted rand index (ARI) comparison across datasets with multiple methods.

As shown in Figure 3, scMGC always achieved maximum ARI values across all datasets, often having a distinct lead relative to other methods. For example, in the Biase dataset, scMGC correctly determined the ground truth cell types with ARI 1.00 while the closest baseline method, FEAST, only achieved ARI 0.84 and highly variable genes (HVG) further went down to 0.544. Once again, in the Pollen dataset, scMGC achieved ARI 0.956 while distinctly outperforming other methods such that none of them achieved higher than 0.82. The superiority of scMGC is even more notable on difficult datasets. Baseline methods returned very low ARI scores (less than 0.3) in Kolod and Goolam in most cases, whereas scMGC significantly improved clustering with ARI 0.58 on Kolod and ARI 0.54 on Goolam. Figure 4 confirms the same phenomenon on NMI: scMGC returned NMI 0.711 on Kolod and 0.721 on Goolam, which is a two‐fold better solution (e.g., feature selection method based on gene expression decomposition [FEED] only returned NMI 0.447 on Kolod). On difficult sets such as Robert, where several methods actually returned near‐random or negative‐worth ARI clustering outputs, scMGC again showed good performance (ARI 0.621, NMI 0.609). On sets where separations were clearer such as Kumar and Chu2, scMGC again led the field, achieving ARI 0.744 and NMI 0.842 on Kumar and a virtually perfect recovery on Chu2 (ARI 0.976, NMI 0.978).

**FIGURE 4:**
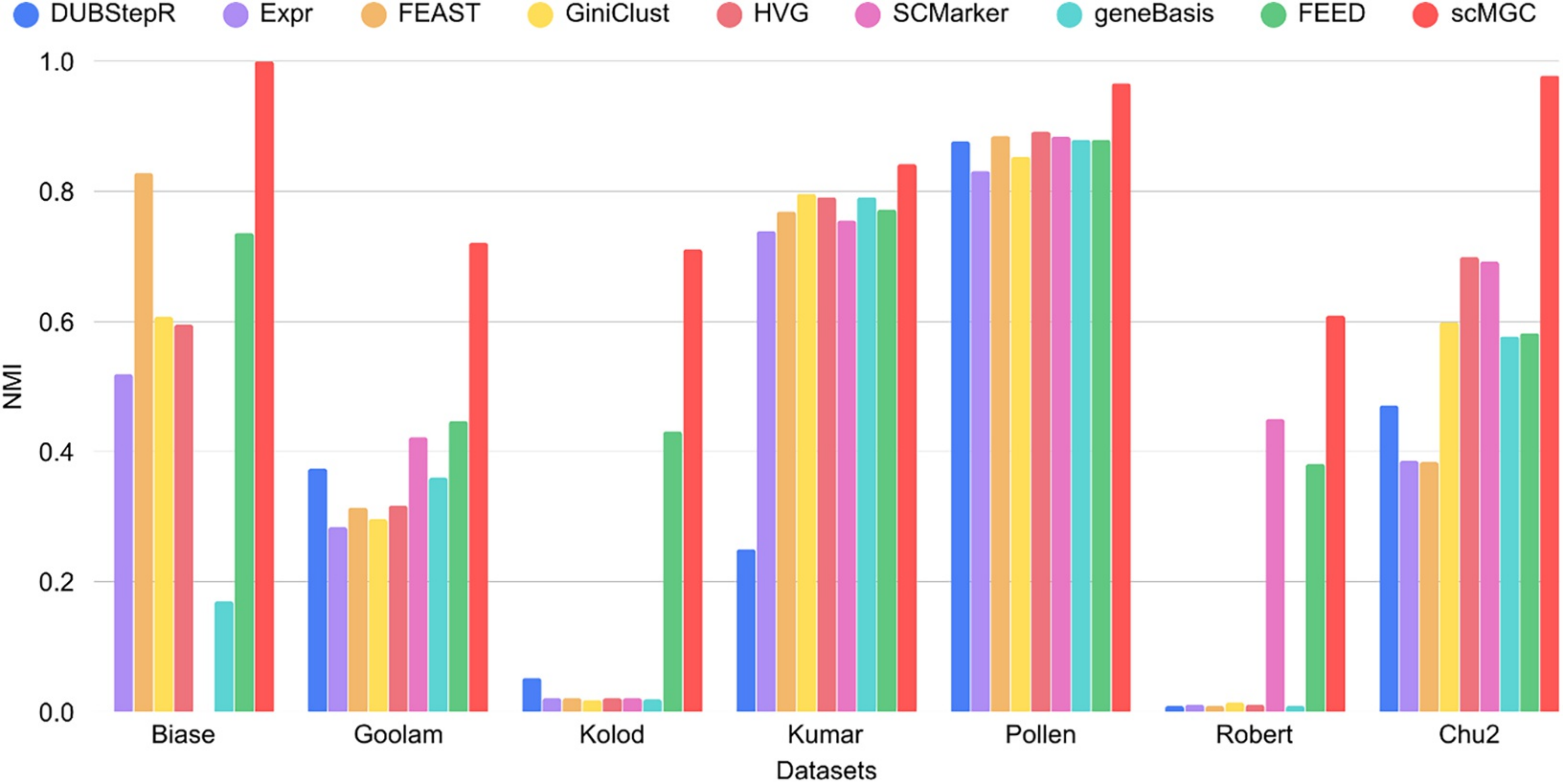
Normalized mutual information (NMI) comparison of single‐cell marker gene clustering with eight competing clustering methods on seven datasets.

Overall, these results as listed in Tables 1 and 2, depicted in Figures 3 and 4 are definitive proof that scMGC is superior to existing methods of clustering on a consistent basis. Overall, scMGC achieved substantial performance gains, yielding weighted average improvements of 31.3% in ARI and 28.2% in NMI relative to the second‐best method, consistently outperforming all competing approaches across datasets.

To further assess scalability beyond small benchmark datasets, we additionally evaluated scMGC on a larger Tabula Sapiens dataset containing 7276 cells. On this dataset, scMGC achieved an ARI of 0.4600 and an NMI of 0.5714, demonstrating stable clustering performance under increased data scale and heterogeneity. These results indicate that the proposed framework maintains robustness when applied to substantially larger scRNA‐seq datasets.

### 2.2 Computational complexity and efficiency

This section presents a comparison of scMGC across the seven datasets with respect to runtime efficiency, memory consumption, and the number of detected genes. This precise computational comparison highlights that the three marker gene detection approaches have significant differences in performance. CellTICS demonstrated remarkable computational efficiency by completing the datasets in time ranges from 0.06 to 0.1406 s while keeping relatively small memory profiles ranging from 12.00 to 152.44 MB. Thus, this makes it highly suitable for rapid initial analyses. COSG showed a medium performance level, with processing times between 0.0625 and 0.2969 s and memory demands between 19.84 and 124.34 MB; thus, it presents a good balance between speed and comprehensiveness.

SMaSH, on the other hand, although having the capability to identify the most marker genes 4261 genes for the Pollen dataset needed a lot more computing power, taking between 3.1875 and 26.3906 s and requiring between 35.04 and 318.10 MB of memory. Its demands were especially high for big datasets, such as Kolod, which took up 25.2188 s and 318.10 MB of memory. The usage patterns seen were directly linked to the dataset size and complexity; small datasets, such as Biase, containing 25,737 genes, permitted real‐time processing times of less than 0.08 s for all methods, whereas intricate datasets, such as Kolod, with 10,685 genes, and Pollen, with 14,805 genes, had a noticeable impact on SMaSH’s computational demands. The results suggest the following guidance for choosing the appropriate method based on particular research goals: CellTICS is preferred for time‐critical exploratory analysis, COSG for research that requires a middle ground of speed and comprehensiveness, and SMaSH for exhaustive investigations where the priority of maximum gene detection makes the added computational burden worthwhile, particularly when working with smaller datasets or when high‐performance computing capabilities are being utilized.

Furthermore, Table 3 compares the computational efficiency of three makers’ identification algorithms: CellTICS, COSG, and SMaSH. CellTICS is the most efficient algorithm with execution times between 0.06 and 0.14 s and the lowest memory usage requirement across all datasets. COSG also displays competitive execution times, often being the best‐performing algorithm on certain datasets such as Goolam and Robert, although it consumes almost twice the amount of random access memory (RAM) needed by CellTICS. On the contrary, SMaSH is the least efficient algorithm, with execution times that are slower by up to two orders of magnitude (e.g., Pollen with 26.39 s) and with the highest memory usage requirement of 318.10 MB. The UMG scoring system seamlessly integrates the three algorithms to optimize their performance and ensure efficient overall execution while performing maker identification with high accuracy. We further examined scalability on the larger Tabula Sapiens dataset (7276 cells). scMGC completed clustering in 4.20 s with a memory usage of 2347.56 MB. For comparison, SMaSH required 89.78 s with 254.66 MB of memory, while COSG required 8.77 s with 4683.91 MB of memory. These results demonstrate that scMGC achieves a favorable balance between runtime efficiency and memory consumption when scaling to larger datasets.

**TABLE 3:** Time and space complexity of marker identification methods.

Dataset	Total genes	Marker genes	Method	Time (s)	Memory (MB)
Biase	25,737	4547	CellTICS	0.08	12.00
			COSG	0.09	19.84
			SMaSH	3.19	35.04
Goolam	41,480	4902	CellTICS	0.14	45.59
			COSG	0.08	80.08
			SMaSH	6.45	141.02
Kolod	10,685	1818	CellTICS	0.08	58.41
			COSG	0.08	115.07
			SMaSH	25.22	318.10
Kumar	22,443	2855	CellTICS	0.06	64.58
			COSG	0.27	124.34
			SMaSH	17.67	209.50
Pollen	14,805	4261	CellTICS	0.11	33.57
			COSG	0.30	57.66
			SMaSH	26.39	156.13
Robert	23,418	2407	CellTICS	0.08	36.11
			COSG	0.06	69.69
			SMaSH	5.16	111.89
Chu2	19,097	4261	CellTICS	0.14	152.44
			COSG	0.30	57.66
			SMaSH	26.39	156.13

### 2.3 Differential expression and GSEA

In addition to validating the biologically meaningfulness of our marker gene‐based clustering for seven datasets, we conducted a differential gene expression analysis using DESeq2 [25], a widely used statistical framework for identifying genes that are significantly differentially expressed between groups by modeling count‐based RNA‐seq data. It identified genes with significant differential expression among cluster groups. The findings indicated highly significant differential expression patterns for many genes at *p* < 0.01, indicating robust transcriptional variation across clusters. As illustrated in Figure 5, there is a clear overlap between the DEG derived from the raw dataset and the marker genes identified by our clustering framework, further supporting the biological consistency and reliability of our method.

**FIGURE 5:**
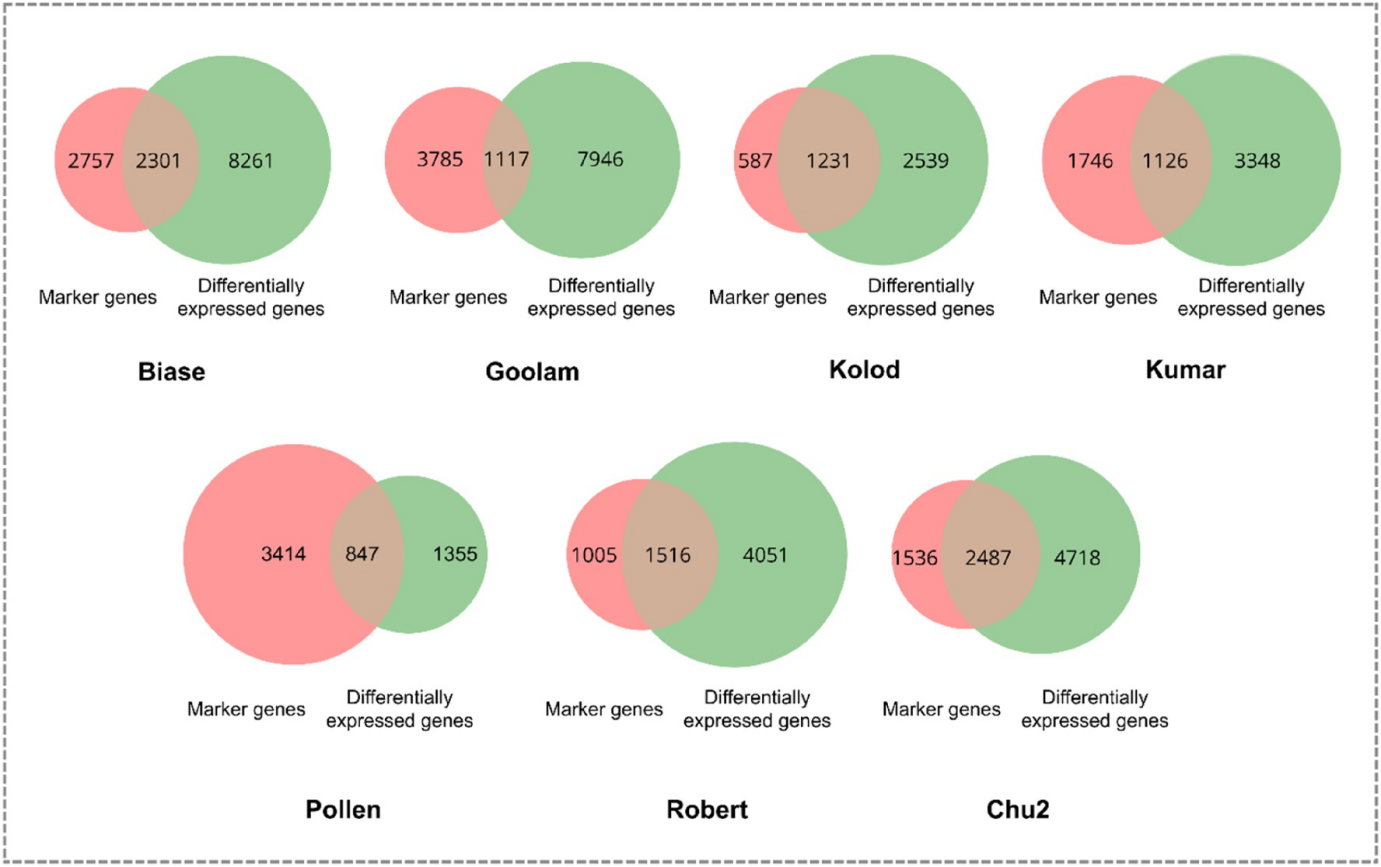
Gene from the differential expression analysis of the raw dataset versus marker gene.

To further investigate the clinical relevance of the identified marker genes, functional enrichment of the identified marker genes was examined with the DAVID bioinformatics platform and the generalized anxiety disorder (GAD) disease enrichment module. The enrichment results are summarized in Table 4. The analysis identified strong associations between the marker genes from the Chu2 dataset and several diseases, such as ovarian cancer, spinal cord injury, breast cancer, type 2 diabetes, and prostate cancer, thus highlighting the clinical relevance of the clusters. A cluster‐level mapping of GAD disease associated genes onto Leiden clusters was performed to examine their expression distributions across cells. The analysis revealed structured cluster‐specific enrichment patterns rather than uniform expression across all clusters, indicating that disease signals arise from defined transcriptional subpopulations. Detailed visualizations and comprehensive interpretations of these patterns, including cluster‐ordered heatmaps, hierarchical clustering, and dot‐plot analyses, are presented in Supporting Information S1: Section S2 with Figures S2–S5.

**TABLE 4:** Generalized anxiety disorder disease table.

Disease	Count	*p*‐value
Ovarian cancer	11	8.9 × 10^−5^
Diabetes, type 2	8	1.9 × 10^−2^
Oral cancer	3	2.2 × 10^−2^
Kidney	3	2.3 × 10^−2^
Prostate cancer	8	3.5 × 10^−2^

In parallel, we utilized WikiPathways to explore enriched immune and signaling‐related disease pathways, thereby gaining deeper functional insights into the biological processes of the clusters. The pathway enrichment results are presented in Table 5. Among the enriched pathways, pleural mesothelioma exhibited the most significant association ($p=1.7\times 10^{-5}$), followed by spinal cord injury and breast cancer pathways. These findings reinforce the disease‐mapping capability of our marker gene‐based clustering strategy and demonstrate its improved specificity compared to the unfiltered dataset. The primary objective of gene set analysis is to determine whether high‐degree genes are significantly associated with predefined biological pathways. Our marker gene‐based clustering strategy demonstrated enrichment for both disease and pathway‐level associations, with increased specificity compared to the unfiltered dataset. Similarly, WikiPathways disease gene sets were projected onto Leiden clusters to assess pathway‐level specificity. The results demonstrate non‐random cluster‐specific enrichment of disease‐related signaling pathways, further supporting the biological coherence of the identified clusters. Detailed cluster‐wise visualizations and quantitative analyses of these pathway enrichments are provided in Supporting Information S1: Section S2 with Figures S6 and S7.

**TABLE 5:** WikiPathways disease table.

Disease	Count	*p*‐value
Pleural mesothelioma	11	1.7 × 10^−5^
Spinal cord injury	5	1.3 × 10^−2^
Breast cancer pathway	5	3.1 × 10^−2^
Endometrial cancer	3	8.4 × 10^−2^
Cancer pathway	8	9.3 × 10^−2^

To validate these findings, gene set enrichment analysis (GSEA) was conducted on the top high‐degree gene counts retrieved from all seven datasets. Genes identified with an false discovery rate (FDR) <0.05 were tested, thereby allowing detection of crucial biological and molecular pathways enriched in each cell type, thereby identifying differences between highly correlated cell families.

### 2.4 Performance on rare and closely related cell types

In the rare cell type scenario, extremely low‐abundance populations such as oocytes (0.23%), spermatogonia (0.9%), and male germ cells (1.6%) were evaluated separately. scMGC achieved strong clustering performance on rare populations, with an ARI of 0.9447 and an NMI of 0.9250, indicating accurate identification despite severe class imbalance. The silhouette score of 0.2073 reflects the expected compactness limitation of rare populations while maintaining clear cluster separation.

To assess fine‐grained discrimination, we applied scMGC to closely related germ cell developmental stages (spermatogonium, spermatocyte, and spermatid). These cell types represent consecutive stages of spermatogenesis and are known to share overlapping transcriptional programs. scMGC achieved an ARI of 0.7163 and an NMI of 0.6716, demonstrating effective separation among highly similar cell states. Notably, the silhouette score reached 0.4570, indicating strong structural separation in the embedding space despite biological similarity.

## 3 DISCUSSION

In this study, we presented scMGC, a unified framework that represents a significant advance in marker gene‐based clustering of scRNA‐seq data. scMGC resolves the fundamental obstacles of heterogeneity, sparsity, and noise in scRNA‐seq data by incorporating a denoising autoencoder and graph‐based clustering with multiple marker gene selection strategies. A central innovation of our approach is the UMG scoring system, which adaptively combines the strengths of individual marker selection methods to yield a stable and biologically reliable feature set.

Across seven benchmark datasets, scMGC consistently achieved higher ARI and NMI scores than competing methods, with particularly strong advantages on more difficult cases such as Kolod, Goolam, and Robert. These datasets frequently produce near‐random or unstable outputs when using baseline methods, but scMGC maintained stable clustering performance. On cleaner datasets such as Pollen, Kumar, and Chu2, scMGC preserved its superior accuracy, confirming both robustness and generalizability. Beyond numerical performance, scMGC’s clusters were biologically validated. The discovered marker genes correlate with significant biological pathways and disease associations, such as breast cancer, type 2 diabetes, and ovarian cancer, according to differential expression and enrichment analyses. This demonstrates that scMGC not only improves clustering accuracy but also produces interpretable results with clinical relevance. From a robustness perspective, rare cell populations and closely related developmental states represent two major challenges for single‐cell clustering methods. The strong performance of scMGC on extremely rare cell types demonstrates its robustness to severe class imbalance. Moreover, its ability to separate closely related germ cell stages highlights its sensitivity to subtle transcriptional differences along developmental trajectories. Together, these results indicate that scMGC is well suited for both coarse‐grained cell type identification and fine‐grained resolution of continuous biological processes. Although the framework shows strong performance, some limitations remain. The current evaluation primarily focuses on benchmark datasets with available ground‐truth annotations. Although we additionally validated scMGC on a larger dataset containing 7276 cells, experiments on ultra‐large datasets (e.g., >100,000 cells) were not conducted due to computational resource constraints. Future work will extend scalability validation to such large‐scale single‐cell atlases to further demonstrate the efficiency of the framework in modern high‐throughput settings. Furthermore, our marker selection module (SMaSH, COSG, and CellTICS) requires pre‐defined cell type labels to identify marker genes. In fully unsupervised scenarios, an initial clustering step would be required to generate pseudo‐labels before applying the scMGC framework. In addition, scMGC currently relies solely on transcriptomic information; future extensions may incorporate multi‐omics or trajectory‐based analyses to further enhance biological insights.

Overall, scMGC offers a robust and interpretable solution for marker gene‐based clustering of scRNA‐seq data, contributing both methodological improvements and biological insight to single‐cell genomics research. The integration of denoising autoencoders with a unified multi‐method marker selection strategy addresses long‐standing challenges of sparsity, noise, and inconsistent feature selection that have hindered single‐cell analysis. The UMG scoring system, by adaptively weighting each marker selection method based on clustering performance, ensures that the most biologically informative genes drive downstream analysis, enhancing both accuracy and interpretability. As single‐cell technologies continue to generate data of increasing scale and complexity, frameworks such as scMGC that combine computational rigor with biological grounding will be essential for extracting meaningful insights from the next generation of transcriptomic studies.

## 4 CONCLUSION

This study presents scMGC, a deep learning framework designed to improve the clustering of scRNA‐seq data through the integration of denoising, marker gene selection, and graph‐based community detection methods. The UMG scoring system is fundamental to the framework, as it adaptively integrates various marker selection strategies to generate stable and biologically reliable feature sets. In evaluations across seven benchmark datasets, scMGC demonstrated superior clustering accuracy compared to established baseline methods. The performance advantage was particularly evident in sparse and heterogeneous datasets, where current methods encountered difficulties in recovering coherent cell populations. In addition to numerical metrics, downstream differential expression and enrichment analyses validated that the identified clusters align with significant pathways and disease associations, thereby reinforcing the computational and biological value of the method. The framework demonstrates scalability and computational efficiency, rendering it suitable for extensive single‐cell investigations. Future extensions may include multi‐omics integration, trajectory inference, or transfer learning to enhance its scope. Overall, scMGC offers a robust and interpretable solution for marker gene‐based clustering of scRNA‐seq data, contributing both methodological improvements and biological insight to single‐cell genomics research.

## 5 MATERIALS AND METHODS

### 5.1 Dataset

In this study, we made use of seven scRNA‐seq datasets. All of these datasets are openly accessible from the National Center for Biotechnology Information (NCBI) and the European Bioinformatics Institute (EMBL‐EBI). Specifically, we included datasets from Refs. [26, 27, 28, 29, 30]. These datasets span a wide range of biological systems, including early embryonic cells, neuronal populations, and pluripotent stem cells. A detailed overview of each dataset’s cell counts, gene coverage, species, and accession IDs is provided in Table 6. To evaluate the scalability of scMGC beyond small benchmark datasets, we further conducted experiments on a larger dataset from the Tabula Sapiens atlas [33]. This dataset contains 7276 single cells derived from human tissues. Tabula Sapiens represents a comprehensive human cell atlas comprising over 1.1 million cells across 28 organs. For our evaluation, we selected a subset to assess clustering robustness under increased data scale and heterogeneity.

**TABLE 6:** Summary of benchmark scRNA‐seq datasets considered for this analysis.

Dataset	Cells	Genes	Types	Species	Accession
Pollen [26]	301	13,678	11	*H*. *sapiens*	SRP041736
Kolod [27]	704	10,685	3	*M*. *musculus*	E‐MTAB‐2600
Kumar [31]	246	45,159	3	*M*. *musculus*	GSE60749
Biase [28]	56	9432	4	*M*. *musculus*	GSE57249
Robert [32]	192	23,418	2	*M*. *musculus*	GSE74923
Goolam [29]	16	9042	2	*M*. *musculus*	E‐MTAB‐3321
Chu2 [30]	1018	19,097	2	*H*. *sapiens*	GSE75748

### 5.2 Method overview

This study introduces the scMGC model, a deep learning framework designed for clustering scRNA‐seq data, with a focus on marker genes. The pipeline integrates a denoising autoencoder, employs multi‐method marker gene selection, and uses clustering to identify biologically significant cell subpopulations.

Figure 6A illustrates the preprocessing of the raw gene expression matrix, which includes quality filtering, normalization, and log transformation. Gaussian noise is introduced to replicate real‐world sparsity through a binomial dropout function (Figure 6B). The denoising autoencoder is trained with a custom loss function that treats zero and non‐zero values differently. This enables the model to learn a compact latent representation that preserves key gene expression features. Besides denoising, Figure 6C presents a multi‐method marker selection module designed to identify key genes that characterize cellular heterogeneity. This module integrates three unique approaches: SMaSH, COSG, and CellTICS. Each of these methods uses distinct techniques, such as Shapley value attribution, scoring dependent on cosine similarity, and feature selection based on pathways. It also uses a weighted scoring mechanism to select the top *n* marker genes from the list as the UMG set for clustering as shown in Figure 6C. In the end, Figure 6D shows the clustering and downstream analysis, where a graph‐based clustering algorithm is applied to a cell similarity network. Moreover, DEA and GSEA are used to validate the biological significance of the clusters. DEA is used to identify the DEG that define each cluster and to reveal potential disease associations. GSEA is used, which maps these genes to known biological pathways. By integrating denoising autoencoders and multi‐method marker gene selection into our method. It also incorporates graph‐based clustering and functional enrichment analysis. By combining these elements, the model provides a scalable and high‐accuracy framework. This results in a biologically interpretable approach to single‐cell transcriptomic clustering.

**FIGURE 6:**
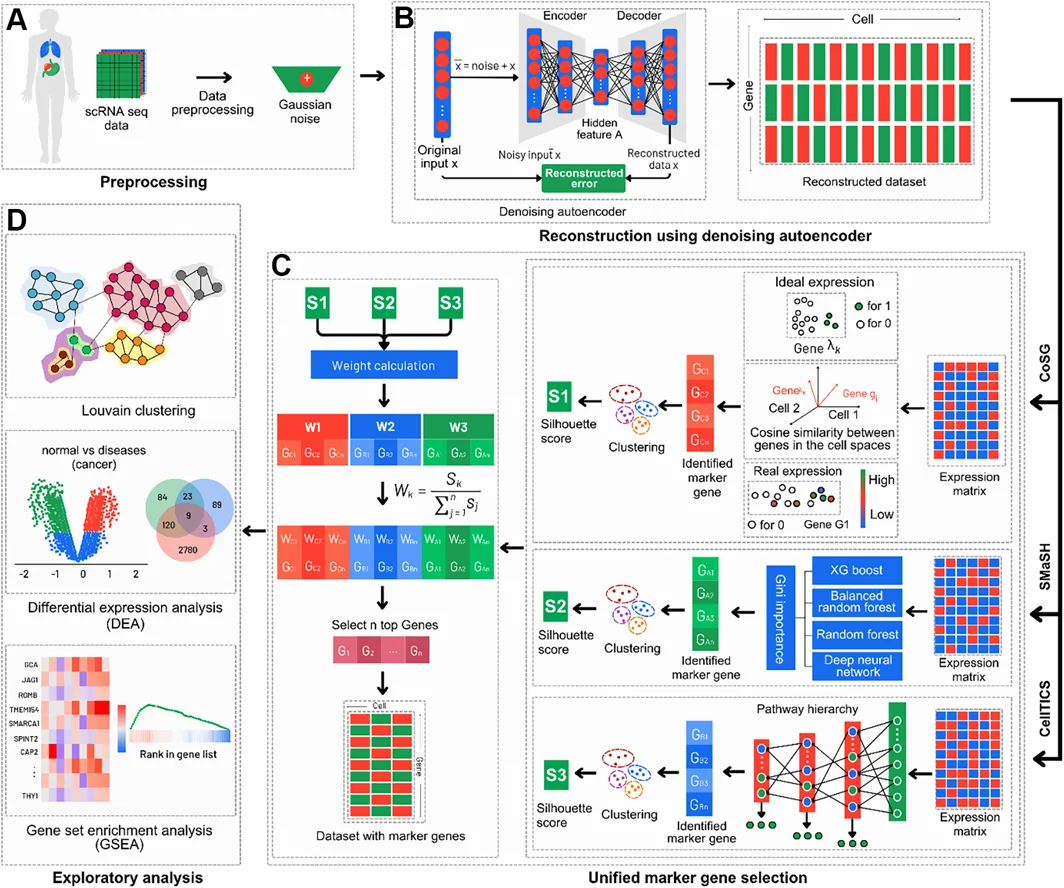
Workflow of single‐cell marker gene clustering. (A) Data preprocessing: filter cells and genes, normalize and logarithmize the expression values. (B) Denoising: incorporate Gaussian noise, train the autoencoder, and denoise the expression values. (C) Marker gene identification and unified marker gene: utilize three cutting‐edge methods to identify marker genes. Each provides a list of marker genes. Genes are combined and scored accordingly from the lists of marker genes based on the weights. The highest *n* number of genes are selected as unified marker genes using the maximum score. (D) Clustering and downstream analysis: Graph‐based clustering was conducted only with unified marker genes, and then downstream analysis was performed to verify whether unified marker genes are genuinely associated with diseases.

### 5.3 Preprocessing

Low‐quality cells and genes with negligible expression were removed, a process known to reduce technical noise and mitigate sparsity [34, 35]. Data loading and conversion to the Scanpy AnnData format enabled effective manipulation and visualization. All preprocessing was performed using the Scanpy [36] and Scikit‐learn [37] libraries. Data cleaning filters out low‐quality data, preventing noise from distorting results. Normalization and log transformation ensure consistent data and eliminate sequencing depth biases.

(1) $$X_{\text{normalized}}=\frac{X_{\text{raw}}}{\text{Total}\;\text{sum}\;\text{of}\;\text{counts}\;\text{per}\;\text{cell}}\times \text{scaling}\;\text{factor}$$

The raw gene expression values, *X*_raw_, represent gene activity in each cell. The total sum of counts per cell is the sum of gene expressions in a cell, and a scaling factor (usually 10,000) normalizes the data across cells.

(2) $$X_{\log}=\log_{2}\left(X_{\text{normalized}}+1\right)$$

After *X*_normalized_ as shown in Equation (1), a logarithmic transformation is then applied to stabilize the data and reduce the impact of large variations, resulting in *X*_log_, the log‐transformed values as shown in Equation (2). In this study, each dataset was analyzed individually using a preprocessing workflow designed to minimize technical variation, including quality control filtering, log transformation, and PCA. To mitigate potential batch effects, our pipeline provides optional correction methods, including Harmony, which adjusts PCA embeddings, and batch balanced k‐nearest neighbors (BBKNN), which constructs a batch‐aware neighbor graph for clustering and visualization. Most datasets lack explicit batch metadata, but inspection of PCA and uniform manifold approximation and projection (UMAP) embeddings indicated that variation was primarily driven by biological factors rather than batch, so batch correction was applied only when necessary.

### 5.4 De‐noising

scRNA sequencing data often contain many zero values due to technical issues, called dropouts. To improve data quality, we used an autoencoder model to remove noise and recover missing gene expression values. This method applied the log transformation and standard scaling and added 10% of non‐zero expression values were randomly masked to simulate technical dropout during training. This percentage was chosen as a conservative corruption level that introduces sufficient sparsity to improve model robustness without overwhelming true biological signal, consistent with prior deep learning‐based scRNA‐seq denoising studies reporting effective artificial dropout rates in the range of 5%–20% [38, 39]. The autoencoder compresses and reconstructs the data using layers with ReLU and sigmoid activations, batch normalization, and dropout. The model was trained using the Adam optimizer and a custom loss function that treats zero and non‐zero values differently. This helps reduce the overestimation of missing values.

To remove noise from sparse gene expression data, we use a custom loss function that treats zero and non‐zero values differently. The loss is defined as follows:

(3) $$L_{\text{sparse}}=\frac{1}{n}\sum_{i_{1}}^{n}\left(1\;-\;m_{i}\right){\left(y_{i}\;-\;{\hat{y}}_{i}\right)}^{2}+\lambda \sum_{i_{1}}^{n}m_{i}{{\hat{y}}_{i}}^{2}$$

In the Equation (3), *y*_*i*_ is the original (true) gene expression value for the *i*th feature, and ${\hat{y}}_{i}$ is the value predicted by the model. The variable *m*_*i*_ is a binary mask that is equal to 1 if *y*_*i*_ is 0 (a missing or dropped‐out value) and 0 otherwise. The first term calculates the mean squared error (MSE) only on non‐zero values, helping the model learn accurate reconstruction. The second term penalizes large predicted values at positions where the input was originally zero, preventing overestimation. The parameter *n* is the total number of features and λ is a small constant (set to 0.1) that controls the weight of the penalty for predicting values where there should be none. This loss function helps the model focus on learning from real data while avoiding noise from artificial zeros.

Our method is supported by recent studies using deep learning for denoising scRNA‐seq data [38, 39, 40]. Additional architectural and training details of the denoising autoencoder are provided in Supporting Information S1: Section S1 along with Figure S1.

### 5.5 Marker gene identification

Three distinct methods were integrated to identify biologically significant marker genes. Because of the different approaches, these methods have the ability to capture unique biological characteristics that ensure robust and comprehensive marker selections.

#### 5.5.1 COSG

COSG is employed to identify suitable marker genes for predefined cell groups [15]. It requires normalized input data and then constructs the gene expression matrix $X\in R^{N\times M}$, In this context, *N* refers to the number of cells, and *M* denotes the total detected genes. The cosine similarity between $g_{i}$ and $\lambda_{k}$ is computed as shown in Equation (4):

(4) $$\cos\left(g_{i},\lambda_{k}\right)=\frac{g_{i}\;\cdot \;\lambda_{k}}{\left\|g_{i}\right\|\times \left\|\lambda_{k}\right\|}=\frac{\sum_{j=1}^{N}x_{ji}\lambda_{jk}}{\sqrt{\sum_{j=1}^{N}{x_{ji}}^{2}}\times \sqrt{\sum_{j=1}^{N}{\lambda_{jk}}^{2}}}$$

To determine whether $g_{i}$ is an effective marker gene for the group $G_{k}$, the COSG score is calculated as follows:

(5) $$\text{COSGscore}\left(g_{i},G_{k}\right)=\cos\left(g_{i},\lambda_{k}\right)\times \frac{{\cos\left(g_{i},\lambda_{k}\right)}^{2}}{{\cos\left(g_{i},\lambda_{k}\right)}^{2}+\mu \sum_{t\in \left\{1,\text{…},K\right\},t\neq k}{\cos\left(g_{i},\lambda_{t}\right)}^{2}}$$

Here, the first term $\cos\left(g_{i},\lambda_{k}\right)$ in Equation (5) measures the similarity between gene $g_{i}$ and the ideal marker gene $\lambda_{k}$ across all cells, while the second term serves as a penalty coefficient. The factor μ (μ≥0) controls the penalization for expression in non‐target cell groups $G_{t}$, t∈{1,…,K},t≠k. A larger μ results in stronger penalization for genes expressed in irrelevant groups.

#### 5.5.2 CellTICS

CellTICS is a feature selection method designed to prioritize marker genes and thereby enhance prediction performance [11]. The measure of high expression for a specific gene g within a given cell type *t* is defined in Equation (6), where $\tau_{g}$ and ${\hat{x}}_{t,g}$ are provided in Equations (7) and (8). Here, n denotes the total number of cell types, and $\exp\;r_{t,g}$ represents the normalized expression value of gene g in cell type t.

To prevent extreme fold changes caused by lowly expressed genes, a pseudo count of 1 is added. As a result, the measure is bounded by $0\leq \phi_{t,g}\leq 1$. This cell‐type‐specificity measure is a refinement of the tissue‐specificity metric introduced by Jiang et al. [41]. A larger $\phi_{t,g}$ value indicates that the gene is more specifically and highly expressed in cell type *t*. For the cell type with maximum expression, $\phi_{t,g}=\tau_{g}$, otherwise $\phi_{t,g}>\tau_{g}$:

(6) $$\varphi_{t,g}=\tau_{g}\hat{x_{t,g}}$$

(7) $$\tau_{g}=\frac{\sum_{t=1}^{n}\left(1-\hat{x_{t,g}}\right)}{n\;-\;1}$$

(8) $$\hat{x_{t,g}}=\frac{{\text{expr}}_{t,g}+1}{\underset{1\leq t\leq n}{\max}\left({\text{expr}}_{t,g}\right)+1}$$

To identify cell‐type‐specific marker genes, we label a gene *g* as a marker for cell type *t* if its score $\phi_{t,g}$ exceeds the 100th quantile of $\left\{\phi_{t,\cdot }\right\}$ within that cell type. Similarly, a gene $g^{\prime }$ is designated a marker for cell type $t^{\prime }$ when $\rho_{t^{\prime },g^{\prime }}$ is greater than the 100th quantile of $\left\{\rho_{t^{\prime }}\right\}$ for that cell type.

#### 5.5.3 SMaSH

SMaSH is a marker gene selection framework based on ensemble learning [42]. Genes are ranked using machine learning classification models such as random forest, balanced random forest, or XGBoost (with XGBoost [16] as the default). The ranking is performed by sorting genes according to their total Gini importance or mean Shapley value, with the latter applied in network mode. From this ranking, a set of relevant markers is produced for each class defined by the user, where the top five markers per class are selected by default.

To ensure robustness, a final heterogeneity check is performed when multiple samples are analyzed, verifying that the selected marker genes are uniformly distributed across at least 70% of the samples included in the dataset. This step increases the reliability of the identified markers by minimizing sample‐specific bias.

### 5.6 UMG

To integrate the strengths of multiple marker gene selection methods, this study proposes the UMG framework. Instead of manually assigning weights to methods, this approach derives method importance scores from clustering performance using silhouette analysis.

Let *g* denotes a candidate gene and k∈{1,2,3,...,n} a marker gene selection method. Each method provides a score $I_{k}\left(g\right)$ representing the importance of gene *g*. The unified score for a gene is computed as a weighted summation, as shown in Equation (9):

(9) $$\text{Score}\left(g\right)=\sum_{k=1}^{n}\omega_{k}I_{k}\left(g\right)$$

Here, $\omega_{k}$ denotes the weight of method *k*, determined through the silhouette coefficient [43]. For a sample *i*, the silhouette score is defined in Equation (10):

(10) $$s\left(i\right)=\frac{b\left(i\right)\;-\;a\left(i\right)}{\max\left\{a\left(i\right),b\left(i\right)\right\}}$$

where a(i) is the mean intra‐cluster distance of *i*, and b(i) is the lowest mean inter‐cluster distance of *i* to another cluster. The average silhouette score for the method *k* is then computed using Equation (11):

(11) $$S_{k}=\frac{1}{m}\sum_{i=1}^{m}s\left(i\right)$$

with *m* being the number of samples. These method‐specific scores are normalized to obtain the final weights, as expressed in Equation (12):

(12) $$\omega_{k}=\frac{S_{k}}{\sum_{j=1}^{n}S_{j}}$$

Using these weights, genes identified by multiple methods are aggregated. Genes not present in a method’s output are assigned zero for that method. The final ranking emphasizes genes consistently supported by high‐weight methods. From this ranked list, the top *N* genes are selected as the UMG set for downstream clustering.

The weighting scheme is applied independently to each dataset. For a given dataset, the method‐specific silhouette scores $S_{k}$ are normalized to obtain the final weights $\omega_{k}$, as defined in Equation (12). This ensures $\sum_{k=1}^{n}\omega_{k}=1$ for that dataset, allowing the UMG score to adaptively reflect the relative clustering performance of each constituent method on the specific data being analyzed.

### 5.7 Clustering algorithm

The Louvain algorithm, originally proposed by Blondel et al. [44], is a widely used and computationally efficient approach for community detection in large‐scale networks. This method relies on optimizing a quality function called modularity. This modularity measures how well a network is partitioned into communities. It performs this measurement by comparing the observed density of edges within communities. This density is then weighed against the density expected in a randomized network. The modularity Q is defined in Equation (13):

(13) $$Q=\left(\frac{1}{2m}\right)\sum \left(A_{ij}-\frac{k_{i}k_{j}}{2m}\right)\;\delta \left(c_{i},c_{j}\right)$$

Here, $A_{ij}$ denotes the adjacency matrix, where $A_{ij}=1$ if an edge exists between nodes *i* and *j*, and 0 otherwise. $k_{i}$ and $k_{j}$ are the degrees of nodes *i* and *j*, *m* is the total number of edges in the network, and $\delta \left(c_{i},c_{j}\right)$ is an indicator function that equals 1 if nodes *i* and *j* belong to the same community and 0 otherwise.

The procedure consists of two iterative steps. Initially, every node is assigned to its own community. Subsequently, each node is evaluated for reassignment to the community of one of its neighbors, and the move is accepted when it yields the maximum improvement in modularity. The change in modularity ΔQ due to moving a node *i* into a community *C* is computed as shown in Equation (14):

(14) $$\Delta Q=\left[\frac{\Sigma_{\text{in}}+2k_{i,in}}{2m}\;-{\left(\frac{\Sigma_{\text{tot}}+k_{i}}{2m}\right)}^{2}\right]-\left[\frac{\Sigma_{\text{in}}}{2m}\;-{\left(\frac{\Sigma_{\text{tot}}}{2m}\right)}^{2}\right]$$

In this expression, it $\Sigma_{\text{in}}$ is the sum of the weights of the edges within community *C*, and $\Sigma_{\text{tot}}$ is the total weight of the edges connected to nodes in *C*. $k_{i}$ is the degree of node *i*, and $k_{i,\text{in}}$ is the sum of the weights from the node *i* to nodes in the community *C*.

In the second phase, a new graph is constructed where each community identified in the first phase becomes a single node. Edges between these new nodes are weighted based on the total weight of edges between the corresponding communities. These two phases are repeated on the reduced graph until no further increase in modularity is possible. This hierarchical and greedy approach allows the Louvain algorithm to scale efficiently to large networks while producing high‐quality community structures.

### 5.8 Downstream analysis

Downstream analysis was performed to derive biological insights from the grouped cells. This involved identifying DEG across clusters and analyzing enriched biological pathways to interpret functional differences among cell populations.

#### 5.8.1 DEA

DEA was carried out to identify genes whose expression levels significantly vary between distinct cell clusters. Expression counts were obtained using a negative binomial distribution, which effectively accounts for variability in sequencing depth and biological dispersion [45].

For each gene, the $\log_{2}$(fold change) in expression between clusters was computed as shown in Equation (15):

(15) $$\log_{2}\text{FC}=\log_{2}\left(\frac{X_{i}}{X_{j}}\right)$$

where $X_{i}$ and $X_{j}$ represent the normalized mean expression levels in two respective clusters. Statistical significance was determined using Wald tests, and multiple testing correction was performed using the FDR method. Genes with adjusted *p*‐values below 0.05 were considered significantly differentially expressed [46].

#### 5.8.2 GSEA

GSEA was applied to explore the biological significance of the DEG. GSEA evaluates whether a predefined set of genes is non‐randomly distributed across a ranked list based on expression changes. An enrichment score is calculated to indicate how strongly a gene set is concentrated near either the top or bottom of the ranked list. The score is then normalized, and its statistical significance is estimated via permutation testing. Gene sets with FDR‐adjusted *p*‐values below a chosen threshold were identified as significantly enriched, revealing key pathways and functional annotations associated with specific cell populations [47].

### 5.9 Robustness analysis on rare and closely related cell types

To evaluate the robustness of scMGC under challenging biological scenarios, we conducted two additional analyses: (i) rare cell type detection and (ii) discrimination of functionally closely related cell types. Rare cell types were defined as those comprising less than 5% of the total cell population. For closely related cell types, we focused on germ cell developmental stages (spermatogonium, spermatocyte, and spermatid), which originate from the same lineage and exhibit highly similar transcriptional profiles [25]. For each scenario, scMGC was applied independently, and clustering performance was evaluated using ARI, NMI, and silhouette score.

## AUTHOR CONTRIBUTIONS

**Shahriar Rahman Niloy:** Conceptualization; formal analysis; investigation; methodology; validation; writing—original draft. **Toushif Muktashid Hasan:** Formal analysis; investigation; methodology; validation; visualization; writing—original draft. **Md. Saiduzzaman Apu:** Formal analysis; investigation; methodology; validation; visualization; writing—original draft. **Fahim Hafiz:** Formal analysis; investigation; supervision; validation; writing—review and editing. **Salekul Islam:** Formal analysis; supervision; validation; writing—original draft. **Riasat Azim:** Conceptualization; formal analysis; methodology; supervision; validation; writing—review and editing.

## CONFLICT OF INTEREST STATEMENT

The authors declare no conflicts of interest.

## ETHICS STATEMENT

This study is a computational analysis of publicly available scRNA‐seq datasets and does not involve the collection of new human or animal data. All datasets used in this study were obtained from established public repositories (NCBI and EMBL‐EBI) and were previously collected and reported under the ethical approvals of their respective original studies. Therefore, no additional ethical approval was required for this work.

## Supporting information

Supporting Information S1

## Data Availability

The code and datasets used in this study are publicly available at GitHub website (srniloy/scMGC). All seven benchmark scRNA‐seq datasets are openly accessible from the national center for biotechnology information (NCBI) and the European bioinformatics institute (EMBL‐EBI) under the accession IDs provided in Table 6 (SRP041736, E‐MTAB‐2600, GSE60749, GSE57249, GSE74923, E‐MTAB‐3321, and GSE75748). The larger Tabula Sapiens dataset used for scalability evaluation is publicly available through the original publication [25].
