# Supplementary material for: Single‐cell marker gene clustering: A unified deep learning framework for marker gene‐based clustering of single‐cell RNA‐sequencing data
Source: Quant Biol. 2026 Jul 24;14(3):e70047. doi: 10.1002/qub2.70047 (PMC13642092; doi:10.1002/qub2.70047)
Supplement: Supplementary file 1 [file QUB2-14-e70047-s001.docx]

**Supplementary Document**

**Single‐cell marker gene clustering: A unified deep learning framework for marker gene‐based clustering of single‐cell RNA‐sequencing data**

Shahriar Rahman Niloy^1^, Toushif Muktashid Hasan^1^, Md. Saiduzzaman Apu^1^, Fahim Hafiz^1^, Salekul Islam^2^, Riasat Azim^1*^

^1^Department of CSE, United International University, Dhaka, 1212, Bangladesh

^2^Department of ECE, North South University, Dhaka,1229, Bangladesh

* Riasat Azim. Tel: +8801796619200; Email: riasat@cse.uiu.ac.bd

# S1. Denoising Autoencoder Architecture and Training Details

To mitigate technical noise and dropout effects inherent in single-cell RNA sequencing (scRNA-seq) data, scMGC employs a denoising autoencoder (DAE) as a preprocessing module. This module aims to restore corrupted gene expression values while preserving biologically meaningful signals prior to downstream analysis.

## Data Preprocessing

The input to the denoising autoencoder is the gene expression matrix stored in $adata.X$. If the matrix is in sparse format, it is converted to a dense representation to enable neural network training. The data are first log-transformed using a natural logarithm transformation $ln(1+x)$ to reduce skewness and stabilize variance. Subsequently, gene-wise standardization is applied using z-score normalization to ensure zero mean and unit variance across genes, improving numerical stability during optimization.

## Noise Injection Strategy

To simulate biological dropout commonly observed in scRNA-seq experiments, sparse noise is artificially introduced into the standardized input matrix during training. Specifically, a binary dropout mask is sampled from a Bernoulli distribution with a dropout probability of 0.1. Expression values corresponding to mask entries equal to one are set to zero, generating a corrupted input matrix. This denoising objective forces the autoencoder to learn robust representations capable of reconstructing missing or masked expression values.

## Autoencoder Architecture

The denoising autoencoder follows a fully connected, symmetric encoder–decoder architecture. Let G denote the number of genes after preprocessing.

**Figure S1.** Detailed encoder–decoder architecture of the denoising autoencoder used for marker gene feature learning.

**Encoder:**

- Input layer: G neurons

- Dense layer with 512 neurons and ReLU activation

- Batch normalization layer

- Dropout layer with a dropout rate of 0.2

- Latent layer with 128 neurons and ReLU activation

**Decoder:**

- Dense layer with 512 neurons and ReLU activation

- Batch normalization layer

- Dropout layer with a dropout rate of 0.2

- Output layer with G neurons and sigmoid activation

This architecture enables nonlinear feature extraction while controlling overfitting through batch normalization and dropout.

## Sparse-Aware Loss Function

Standard reconstruction losses are insufficient for scRNA-seq data due to the ambiguity of zero values. To address this, a custom sparse-aware mean squared error loss is employed. The loss separately penalizes reconstruction errors for non-zero and zero-valued entries. Errors on non-zero values are fully penalized to preserve true expression levels, while predictions at positions corresponding to zero entries are penalized with a lower weight (0.1) to discourage artificial inflation of dropout values.

## Training Configuration

The autoencoder is trained using the Adam optimizer with a learning rate of 0.001. Training is conducted for up to 300 epochs with a batch size of 256. The dataset is split into training and validation sets using an 80:20 ratio. Early stopping is applied based on validation loss with a patience of 15 epochs, and the best-performing model weights are restored. Additionally, a ReduceLROnPlateau scheduler is used to reduce the learning rate by a factor of 0.5 if the validation loss does not improve for 5 consecutive epochs.

## Output Generation

After training, the autoencoder is applied to the noisy input data to generate a denoised gene expression matrix. The standardized output is then inverse-transformed to restore the original expression scale. The denoised matrix replaces the original $adata.X$ and is used for subsequent marker gene identification and downstream analyses.

# S2. Disease Association from marker genes

We appreciate the reviewer’s comment that the connection between GAD and WikiPathways disease associations (Table 4 and 5 for the chu2 dataset) and specific clusters could be clearer. To address this, we systematically mapped disease-associated genes onto clusters and visualized their distributions across cells. The cluster-ordered heatmap (Figure S2) shows that disease genes are not uniformly expressed but instead concentrate within specific clusters, indicating that disease signals arise from defined cellular subpopulations. For example, several ovarian cancer–associated genes such as *FN1, IFI16,* and *IL1R1* display strong enrichment in cluster 2, while *GSTP1* and *FN1* extend into cluster 3, and *IGFBP5* and *GSTP1* appear in a subset of cluster 7. This pattern indicates that the ovarian cancer signature is primarily driven by cluster 2 with secondary contributions from transcriptionally related neighboring clusters. The same figure shows that diabetes-associated genes, including *LRP6, MEIS1,* and *DLC1*, are strongly concentrated in cluster 4, whereas *CXCR4* and *DLC1* are enriched in cluster 3, suggesting that diabetes-related pathways are partitioned across multiple but related cellular states rather than restricted to a single cell population.

**Figure S2.** Heatmap of disease-associated gene expression across Leiden clusters.

Figure S2. Shows the Heatmap of disease-associated gene expression across Leiden clusters. Cells are ordered by cluster identity (0–7), showing cluster-specific enrichment patterns; several cancer- and metabolic-related genes concentrate strongly in different clusters.

The hierarchical heatmap with dendrogram (Figure S3) further supports these relationships by grouping clusters according to similarity in disease-gene expression profiles. Clusters 2 and 7 branch closely together, consistent with their shared enrichment of cancer-associated genes such as *ANXA2, CTSB,* and *GSTM3*, while clusters 4 and 6 form another branch characterized by transcriptional regulators such as *MEIS1/2* and signaling receptors, including *LRP6*. In contrast, clusters 0 and 1 cluster together due to their relatively low expression of most disease genes, indicating that they represent baseline or less disease-relevant cellular states. This hierarchical structure demonstrates that disease signals are not random but organized along transcriptional similarity axes that reflect biologically meaningful relationships among clusters.

**Figure S3.** Hierarchically clustered heatmap of the same gene set across Leiden clusters. The dendrogram highlights transcriptional similarity among clusters.

The dot-plot visualization (Figure S4), which simultaneously displays the fraction of expressing cells and mean expression level, provides quantitative confirmation of these trends. Cluster 2 shows both high expression intensity and large expressing fractions for *ANXA2, FN1,* and *IGFBP5*, reinforcing its role as a major hub for ovarian and prostate cancer–related genes. Cluster 4 shows the highest fractions for *MEIS1* and *LRP6*, consistent with a diabetes-associated transcriptional state. Cluster 3 exhibits prominent expression of *HRAS* and *CXCR4*, linking it to oral cancer and metabolic signaling pathways. Cluster 7 stands out for strong *GSTM3*, suggesting a stress-response to cancer progression. Because the dot size reflects the percentage of cells expressing each gene, these patterns demonstrate that disease associations are driven by substantial subsets of cells rather than small outliers.

**Figure S4.** Dot plot summarizing mean expression (color) and fraction of expressing cells (dot size) for disease-associated genes in each cluster.

The scaled expression dot plot (Figure S5) confirms that these patterns persist after normalization, indicating that the observed enrichments are not artifacts of raw expression magnitude. In this representation, clusters 2 and 7 remain enriched for multiple cancer genes, cluster 4 retains strong diabetes-related signals, and cluster 3 continues to show mixed oncogenic and signaling signatures. Importantly, several genes appear across more than one cluster, such as *DLC1*, which is associated with both diabetes and prostate cancer and is expressed in clusters 3 and 4. This overlap suggests that certain molecular pathways are shared across diseases but are deployed in different cellular contexts. Thus, the figures collectively demonstrate that disease genes form structured, interpretable patterns across clusters rather than diffuse expression.

**Figure S5.** Scaled dot plot of disease gene expression across clusters. After normalization, cluster-specific enrichment patterns remain evident, confirming that disease associations arise from defined cellular subpopulations rather than global expression differences.

Taken together, these results clarify that each disease signature maps onto distinct but sometimes overlapping cellular populations. We can summarise our observations for GAD diseases findings:

- Disease genes show clear cluster-specific enrichment rather than uniform expression.
- Cluster relationships in the dendrogram confirm that disease-linked clusters are transcriptionally similar.
- Overlapping gene expression across clusters reflects biological heterogeneity rather than noise.

Furthermore, for Table 5, we mapped WikiPathways disease gene sets onto Leiden clusters and examined their expression patterns. The clustered heatmap (Figure S6) shows that disease genes display structured, cluster-specific enrichment rather than uniform expression. For instance, several Pleural Mesothelioma genes, such as *CTNNB1* and *LRP6,* are strongly enriched in clusters 3 and 4, while *DKK1* and *IL1R1* show localized peaks in cluster 2. Spinal Cord Injury genes also concentrate non-randomly, with *IL1R1* and *MMP9* enriched in cluster 2 and signaling regulators such as *CDC42* and *RHOB* showing stronger expression in clusters 3–4. The dendrogram in Fig. 1 further supports this organization, as clusters 3 and 4 group together due to shared oncogenic and signaling signatures, whereas clusters 0 and 1 branch separately and exhibit comparatively low disease-gene expression, indicating baseline-like cellular states.

**Figure S6.** Hierarchically clustered heatmap of WikiPathways disease–associated genes across Leiden clusters. Cells are ordered by cluster identity, and the dendrogram highlights transcriptional relationships among clusters, revealing cluster-specific enrichment of disease genes.

The dot plot (Figure S7) quantitatively confirms these trends by showing both the expression level and fraction of expressing cells. Clusters 3 and 4 display strong signals for cancer-related genes, including *HRAS* and *CTBP1*, identifying them as major hubs for oncogenic pathways. Cluster 2 shows the strongest representation of injury-response genes, such as *IL1R1,* linking it specifically to the Spinal Cord Injury pathway, while cluster 6 shows elevated *PDPK1*, consistent with Endometrial Cancer pathway enrichment. Core signaling genes like *BRAF, BAK1,* and *HRAS* appear across multiple clusters, reflecting shared regulatory modules active in distinct cellular contexts. Overall, these results demonstrate that WikiPathways disease signatures correspond to defined transcriptional subpopulations rather than diffuse expression across all cells.

**Figure S7.** Dot plot summarizing mean expression (color) and fraction of expressing cells (dot size) for WikiPathways disease genes across clusters.
